# Evaluating the level of adherence to Ministry of Health guidelines in the management of Severe Acute Malnutrition at Garissa Provincial General hospital, Garissa, Kenya

**DOI:** 10.11604/pamj.2014.17.214.3821

**Published:** 2014-03-18

**Authors:** Osman Warfa, Daniel Njai, Laving Ahmed, Bashir Admani, Fred Were, Dalton Wamalwa, Boniface Osano, Patrick Mburugu, Musa Mohamed

**Affiliations:** 1 Department of Paediatrics and Child Health, College of Health Sciences, University of Nairobi, Kenya; 2 Garissa Provincial General Hospital, Garissa, Kenya

**Keywords:** Severe malnutrition, adherence, audit, WHO guidelines

## Abstract

**Introduction:**

Half of Kenya's high infant and under five mortality rates is due to malnutrition. Proper implementation of World Health Organization's (WHO) Evidence Based Guidelines (EBG) in management of severe acute malnutrition can reduce mortality rates to less than 5%. The objectives were to establish the level of adherence to WHO guideline and the proportion of children appropriately managed for severe acute malnutrition (steps 1-8) as per the WHO protocol in the management of severe acute malnutrition. This was a short longitudinal study of 96 children, aged 6-59 months admitted to the pediatric ward with diagnosis of severe acute malnutrition.

**Methods:**

Data was extracted from patients’ medical files and recorded into an audit tool to compare care provided in this hospital with WHO guidelines.

**Results:**

Non-edematous malnutrition was the commonest presentation (93.8%). A higher proportion (63.5%) of patients was male. Most (85.4%) of patients were younger than 2 years. Patients with non-edematous malnutrition were younger (mean age for non-edematous malnutrition was 16 (± 10.6) months versus 25 (± 13.7) months in edematous malnutrition). The commonest co- morbid condition was diarrhea (52.1%). Overall, 13 children died giving an inpatient case fatality rate of 13.5%. Appropriate management was documented in only 14.6% for hypoglycemia (step1), 5.2% for hypothermia (step 2) and 31.3% for dehydration (step 3).

**Conclusion:**

The level of adherence to MOH guidelines was documented in 5 out of the 8 steps. Appropriate management of children with severe acute malnutrition was inadequate at Garissa hospital.

## Introduction

Every year 10.6 million children die worldwide due to preventable conditions such as pneumonia, diarrhea, malnutrition, malaria and measles. Of these deaths, malnutrition accounts for about 2.2 million deaths annually in children under the age of 5 years [1, 2]. In Kenya, the levels of chronic malnutrition have remained high with no significant change since 1998. Kenya's North Eastern Province is an arid and semi arid region with prolonged periods of drought from time to time. While the national prevalence of acute malnutrition is 6%, the prevalence in this region is 30%. The infant and under five-mortality rate is higher than the national average, staggering at 57 and 80 per 1000 live births. Malnutrition alone accounts for 50% of Kenya's high infant and under 5 mortality [1, 3]

Because malnutrition is a cause of profound physiological and metabolic changes, a malnourished child responds poorly to treatment and has a 50% chance of dying, even in a hospital setup. As malnutrition is also linked to increased risk of deaths from diarrhea, pneumonia, malaria and measles, it contributes to more than 60% of hospital deaths [4–6]. Global studies have shown that poor hospital care of severely malnourished children contributes to the high case fatality rates [2, 4, 6–11]. In an effort to address the high child mortality, the World Health Organization (WHO) has developed a guideline with ten-step protocol to improve case management for children with severe acute malnutrition (SAM) [12]. In hospitals where the WHO guidelines was introduced and implemented, studies have shown a reduction in mortalities, from high rates of 50% to as low as 6% [2, 6, 8, 12–15].

The use of these evidence-based guidelines in Kenyan hospitals has the potential to reduce Kenya's high infant and under five mortality rates particularly in North Eastern region. We therefore carried out a short longitudinal study with the aim :(1) to determine the proportion of admissions appropriately managed for severe malnutrition using WHO guidelines, (2) to establish the level of adherence to WHO guidelines in the management of severe malnutrition at Garissa Provincial General Hospital (GPGH) in children aged 6-59 months.

## Methods

### Study design, study site, study period and study population

The design was a short longitudinal study at Garissa Provincial General Hospital (GPGH) over a four month period (July to October 2012). The study sites included the pediatric ward (PW), the Maternal and Child Health Clinic (MCH) and the Outpatient department (OPD). Garissa PGH is a regional referral hospital for North Eastern Kenya (population of 2.23 million) and three other neighboring districts. It also serves a further 400,000 refugees from the refugee camps in Daadab. It is a 250 bed-capacity hospital with 297 technical and non-technical staff. Sample size was calculated using the Fisher's formula, n = {t2 x p(1-p)}/m2,

Where n = minimum sample size required, t = confidence level at 95% (standard value of 1.96), p = estimated prevalence of malnutrition in the project area (50% (0.5)) and m= margin of error at 10% (0.01), Our study population of 96 children aged 6-59 months whose guardians gave informed written consent were purposively sampled. Children with chronic conditions (cerebral palsy, cardiac disease and renal disease) that predispose them to severe malnutrition were excluded. Based on the information obtained from the medical records, the management for all children aged 6-59 months who were admitted from OPD and MCH and admitted to PW for severe malnutrition was reviewed using an audit tool so as to determine the proportion of children appropriately managed for the 1st 8 steps of the WHO guideline for the management of severe acute malnutrition. A self-administered questionnaire was also used to collect additional information from health workers on their awareness on availability and accessibility of WHO guidelines, trainings on management of malnourished children and inventory of essential supplies.

### Data collection Methods

The principle investigator (OW) with two research assistants visited the MCH, OPD and Pediatric Ward daily from7am to 10pm and purposively recruited eligible patients. Data extracted from medical file of patients was recorded into an audit tool to compare care provided with guideline recommendation for 1st 8 steps. A self-administered questionnaire was also used to collect additional information from health workers on their awareness on availability and accessibility of WHO guidelines, trainings on management of malnourished children and inventory of essential supplies.

### Audit of Medical Records

The researcher and the assistants audited purposively selected medical records/files for all admitted patients with severe malnutrition using a prepared audit tool. The records extracted included the following:-the clinician's admission notes (to check if there was documentation of the following : Anthropometry measurements (weights and lengths (or height if aged 2 years or older) and weight/height ratio or Z-score and was compared with the median National Center for Health Statistics (NCHS) reference population), presence or absence of edema and wasting, correct classification of malnutrition, presence or absence of diarrhea/ dehydration/shock, conscious level, the treatment sheets(correct choice/dosages of antibiotics, micronutrients, electrolytes, correct choice/volume/route of administration of F75 and F100, and also check if “keep warm” was prescribed), observation charts & nursing cardex ( if temperature was monitored 6-hourly, correct monitoring of fluids and feeds), the laboratory request forms (results of random blood sugar, hemoglobin level and blood slide for malaria). The audit was conducted to check if the 1st 8 steps were correctly applied or not.

### Data management

Data were cross checked for completeness and accuracy before entering into the computer using Microsoft Access. It was analyzed using Stata Version 11. Categorical variables were summarized using proportions and measures of central tendencies (means, medians) and dispersions (standard deviations) used for continuous variables. The outcome was calculated as the percentage of children managed according to the guideline recommendations. The outcome was compared with other categorical variables using the chi square test. Appropriate correlation was applied to the chi square using the Yates or Fishers exact tests when expected cell counts were less than five. T-tests were used to compare means for continuous variables.

## Results

A total of 96 children admitted with diagnosis of severe acute malnutrition were recruited. Table 1 summarizes the demographic characteristics of the participants. Of these, 90 (93.8 %) were admitted with diagnosis of non-edematous malnutrition while 6 (6.2%) were admitted with edematous malnutrition. No patient with marasmic -kwashiorkor was admitted. The average age of participants was 16.6 with a standard deviation of 10.9 months. The mean age for admissions with non-edematous malnutrition was 16 (± 10.6) months compared to a mean age of 25 (± 13.7) months among admissions with edematous malnutrition (difference = 9.0 months, 95% CI -0.01 to 18.0, p value = 0.05). Overall, 85.4% of the subjects were below 24 months of age. The majority of the patients were males 63.5% (n=61) while 36.5% (n =35) were females, giving a male to female ratio of 1.7:1. Of the 90 patients who presented with non-edematous malnutrition, 59 (65.6%) were males and 31(34.4%) were females. The range for WHZ score was -3SD to below -4SD. Fifty (52%) children had Z scores of -3SD to - 4SD while 46 (47.9%) had Z scores < 4SD. There was no significant association of WHZ scores with either patient age (p = 0.17) or gender (p = 0.92). The commonest co-morbid conditions were diarrhea (52.1%), malaria (43.7%) and pneumonia (31.3%). The mean length of hospital stay was 7.6 days (± 2.9) with a range of 1 to 14 days. Overall, 13 died before discharge, giving an inpatient case fatality rate of 13.5%. Out of the 13 deaths, 10 (77%, 95% CI 46-95%) were males while three (23%, 95% CI 5-54%) were females (p = 0.36). Mortality was not significantly associated with the type of malnutrition (P=1.00) or gender (p=0.36) or patient age (mean age alive = 16.5 months versus died = 16.6 months, p = 0.98).

**Table 1:** Demographic characteristics of admissions with acute malnutrition at Garissa Provincial General Hospital

Variable	Category	Frequency (%)
Age in months		
	Below 24 months, n (%)	82(85.4)
Above 24 months, n (%)	14(14.6)
Child's gender	Male	61(63.5)
	Female	35(36.5)
	Total	96 (100)
Average age in months	All children (n = 96)	16.6 (± 10.9)
	Non edematous malnutrition (n = 90)	16 (± 10.6)
	Edematous malnutrition (n = 6)	25 (± 13.7)
	Marasmic- Kwashiorkor (n = 0)	N/A

**Audit of care for severe acute malnutrition at Garissa PGH**: As shown in Table 2 and Table 3, 8 out of the 10 guideline recommended malnutrition management steps were audited. The results of health workers interviewed at GPGH is presented in Table 4 while the summary of availability of essential supplies is shown in Table 5.

**Table 2:** Implementation of malnutrition management steps in children at Garissa Provincial General Hospital

Correct amount of IV Fluids in 1^st^ hour	0/3(0)	1/4 (25)
Correct recording and monitoring of IV fluids	1/3(33.3)	2/4(50)
IVF administered for dehydration ( no shock)	3/59(5.1)	3/62(4.9)
Hb done	59(61.5)	70(72.9)
Hb < 5g/dl	13(22)	15(21.4)
Transfused under iv furosemide	7/13(53.8)	12/15(80)
**Electrolyte imbalance (step 4)**	NA	
Electrolyte imbalance corrected with F75 n=82		82(85.4)
**Infection (step 5) n=96**	NA	
Antibiotics administered		86(89.6)
Mebendazole administered		16(16.7)
**Micronutrient deficiencies (step 6) n=96**	NA	
Vitamin A (received correct dose)		78(81.3)
Folic Acid (received correct dose)		63(65.7)
Multivitamin (received correct dose)		86(89.6)
Iron withheld initially		66(68.8)
Iron given in catch up phase		33(34.4)
**Provide initial feeds for:**		
**Initial stabilization (step 7) n=96**	NA	
F 75 prescribed		88(91.7)
Feed intake monitored daily		82(85.4)
**Catch up growth (step 8) n=96**	NA	
Transition to F100		84(87.5)
Correct Volume of F100 prescribed		81(84.4)
Volume of F100 increased after transition period		84(87.5)

**Table 3:** A Summary of Proportion of Children Appropriately Managed for Steps 1-8

Prevention and treatment:		Appropriate treatment (%)
Step 1	Hypoglycemia	14.6
Step 2	Hypothermia	5.2
step 3	Dehydration	29.1
Step 4	Electrolyte imbalance	85.4
Step 5	Infection	89.6
step 6	Micronutrient deficiencies	81.3
Step 7	Initial stabilization	91.7
Step 8	Catch up growth	87.5

**Table 4:** Results of health workers interviewed at Garissa Provincial General Hospital

Characteristic	Frequency (%)
**Gender**	
Male	14 (43.8)
Female	18 (56.3)
Had any training in the past three months (n=14)	
On malnutrition management	3 (21.4)
Other trainings	11 (78.6)
Had training on Integrated Management of acute malnutrition (n=32)	7 (21.9)
General awareness of existence of MOH guidelines (n=32)	
Guidelines on Integrated Management of Acute Malnutrition	12 (37.5)
Others	20 (62.5)
**Availability of MOH guidelines (n=32)**	
Integrated Management of Acute Malnutrition	12 (37.5)
Other guidelines	20 (62.5)
**Guideline location (n=32)**	
Ward	2 (6.3)
MCH	5 (15.6)
Personal copy	3 (9.4)
Don't know	22(68.8)

**Table 5:** Summary of availability of essential supplies

Availability of	All the time Freq (%)	Part of the time Freq (%)	Rarely- Freq (%)
Weighing Scale/ Height board	31 (96.9)	1 (3.1)	0 (0)
Thermometer	10 (40)	8 (32)	7 (28)
Glucometer/ glucostix	2 (9.5)	6 (28.6)	13 (61.9)
Drugs-Xpen/gentamycin/ metronidazole/ Folate/ Vit A	30 (94)	2 (6)	0 (0)
IV Fluids HSD & 5% dextrose	23 (79.3)	4 (13.8)	2 (6.8)
ReSoMal	29 (90.6)	3 (9.4)	0 (0)
F75	30 (93.7)	2 (6.3)	0 (0)
F100	31 (96.9)	1 (3.1)	0 (0)
Others-Potassium / heaters/ blankets	26 (86.7)	3 (10)	1 (3.3)

***Step 1: Treatment or prevention of hypoglycemia***: Out of the 96 patients, RBS was done in only 1 patient (1.1%) at OPD and an additional 4 (4.2%) patients on admission to the pediatric ward. Four (4.2%) patients at OPD compared to 10(10.5%) in the in-patient received treatment for hypoglycemia (using 5mls/kg of 10% dextrose or Oral /NGT glucose or feeds) according to the clinician's recommendation although RBS was not done in these treated patients. Overall only 5/96(5.2%) patients had a RBS done and 14/96 (14.6%) received treatment. On average, patients were fed 2.6 hours after admission.

***Step 2: Treat / prevent hypothermia***: There are no specially “designed” malnutrition rooms, so children with severe malnutrition were nursed in the general pediatric rooms. Although 28 patients (37.3%) at OPD and 26(32.9%) at in-patient had temperature below 35.5º C, only 11.1% of OPD patients and 10 % of In-patients had “keep warm” prescribed in their treatment sheets. Overall 5/96 (5.2 %) in-patients were provided with “warmth” and none of the patients at OPD. Correct management for hypothermia was done for 5/96 (5.2%) of patients.

***Step 3: Treat or prevent Dehydration*** : Sixty-four out of 96 (66.7%) patients at OPD and 67/96(69.8%) patients in the ward had diarrhea. At OPD, 59 patients had dehydration and five patients had shock. In the ward, 62 patients had dehydration while five patients had shock. Twenty-six (44.1%) patients at OPD and 30(48.4%) patients in the ward with dehydration were treated with ReSoMal. However, correct monitoring of ReSoMal was done for only 2/26 (7.7%) patients at OPD and 13/30 (43.3%) patients in the ward. Of the 10 patients diagnosed with hypovolemic shock, 3/5 (60%) patients at OPD and 4/5 (80%) patients in the ward were treated with correct amount and type of IV fluids (HSD with 5% dextrose). Even so, the correct amount in 1st hour was done for ¼ (25%) of in-patients. Monitoring and recording of IV fluid was done for 1/3(33.3%) and 2/4(50%) of OPD and in-patients respectively. Contrary to the WHO guidelines, six patients not in shock were treated with IV fluids. Therefore, correct management for Step 3 was 31.3% for OPD and 26.9% for IPD.

***Step 4: Correct electrolyte imbalance***: Correction of electrolyte imbalance was done for 55/96 (57.3%) of patients in the ward using commercially prepared F75 that contains potassium.

***Step 5: Treat or prevent infections***: Out of the 96 patients admitted, 86/96(89.6%) received correct dosages of antibiotics (crystalline penicillin/ gentamycin /oral metronidazole) that cover for gram-positive and gram- negative organisms.

***Step 6: Correct micro-nutrient deficiencies***: Out of the 96 patients admitted, 78/96 (81.3%) received correct dose of Vitamin A with 65.7% and 89.6 % receiving folic acid and multivitamins as required. Sixty-six (68.8%) of the in-patients did not receive iron (iron withheld) in the initial phase as required. However, only 34.4% (n=33) of patients received iron in the catch up phase or on discharge.

***Step 7: Initiate feeding***: Out of the 96 patients, 88/96(91.7%) were prescribed and received correct amount and frequency of F75 as per the guidelines. Daily monitoring of feeds was done for 82 (85.4%) patients. The route of feeding was specified as oral for 81/96 (84.4%) of patients and 10/96(10.4%) by NGT tube. On average, children received their first feed 2.6 hours after admission.

***Step 8: Catch up growth***: Out of the 96 patients admitted, 87.5% (n=84) were correctly transited from F75 to F100, with 96.4% (n=81) of them having correct volume prescribed. Eighty-four (87.5%) patients had volume increased after transition period. The initial stabilization phase lasted an average of 3 days (SD ± 0.7) without significant differences (0.14 days, 95% CI -0.7 to 1) between different types of malnutrition (P = 0.69). The rehabilitation phase lasted 5.2 days (SD± 3.9). There was no significant difference (0.3 days, 95%CI -4.5 to 5.1) between the two groups (P=0.87).

### Management for anemia

Hemoglobin level was measured in 59 (61.5%) patients at OPD and 70(72.9%) patients on admission. 22% (n=13) of children at OPD and 21.5% (n=15) of children in the ward had severe anemia (Hb <5g/dl). The mean hemoglobin for both groups was 8.7 g/dl (± 3.5). Among those who had severe anemia, 7/13(53.8%) at OPD and 12/15(80%) of admissions were transfused correctly under intravenous furesemide as per the WHO guidelines. Out of the 13 patients who died, 5 (38.5%) of them had severe anemia with a mean hemoglobin of 3.1g/dl (range 1.3- 4.7g/dl).

## Discussion

Appropriate management of patients with SAM following the 10 step WHO guideline has resulted in substantial improvement in morbidity & mortality in such children. Our study unfortunately documents that adherence to guidelines is inadequate at the provincial general hospital in Northern Kenya. Major deficiencies were noted in treatment and prevention of the following critical steps - hypoglycemia, hypothermia and dehydration despite the availability of essential supplies. There was also delay of up to 2.6 hours in initiating feeds after admission.

Early recognition and anticipation of common problems through a triage system was shown to reduce mortality in Bangladesh [10]. This study showed that a majority (96 %) of the patients were appropriately triaged as staff at the registration desks (OPD & MCH) had training on triage, a finding also noted by Nzioki et al at Kenyatta National Hospital, the main public tertiary referral hospital in Kenya [7]. This is in contrast to the study by Ashworth et al which showed “absence” of triage in South African hospitals with children waiting up to 8 hours before admission [6]. Although severely malnourished children are at increased risk of developing hypoglycemia (RBS <3mmols), appropriate prevention and treatment for hypoglycemia was inadequately done at Garissa hospital. Nzioki et al at Kenyatta National Hospital [7] reported comparable results. Lack of training on WHO guidelines and therefore lack of knowledge about the dangers of hypoglycemia as well as shortage of nursing staff could explain inadequate management for hypoglycemia. Although children with severe malnutrition are highly susceptible to hypothermia, our study showed inadequate prevention and treatment for hypothermia. Patients were nursed in the general pediatric rooms with no “specially-designed” rooms for malnutrition contrary to the WHO guidelines. Furthermore, there were no measures taken to keep the patients warm despite the availability of blankets and heaters. Only a few (11%) mothers were also informed to do so.

The diagnosis of dehydration and shock in the severely malnourished children is quite challenging. In our study, treatment and prevention of dehydration was inadequate and correct monitoring and recording of vital signs for patients on ReSoMal and/or IV fluids to check for over-hydration was not done thereby risking cardiac failure. Contrary to the WHO guidelines, six patients not in shock were treated with IV fluids. Ashworth et al reported similar findings where IV fluids were indiscriminately used in patients with malnutrition and dehydration [2]. Lack of training and therefore lack of knowledge on the dangers of poor monitoring of patients as well as shortage of nurses, may have contributed to our findings. Correction of electrolyte imbalance is an essential component in the management of severe malnutrition. In this study, electrolyte imbalance was corrected in 85% of admissions using commercially prepared F-75 that provides the required extra potassium and magnesium as per the WHO guidelines. Children with severe malnutrition commonly present with serious infection without usual signs of infection such as fever. In our study, routine administration with correct doses of broad-spectrum antibiotics to cover for gram- positive and gram- negative organisms was done for 90% of admissions as per WHO guidelines. The finding is similar to that reported by Nzioki et al where 90% of patients received appropriate antibiotics at Kenyatta National Hospital [7]. Findings by Ashworth et al, showed a lower rate (46%) of antibiotic administration due to doctors reluctance to prescribe antibiotics to patients at Sipetu Hospital [6]. Children with severe malnutrition have vitamin and mineral deficiencies. This may be due to inadequate amount in their diet as well as substantial loss in diarrhea. In this study, high proportion of patients received correct dosages of vitamin (81%), multivitamin (90%) and folic acid (66%). Although iron was correctly withheld in the initial phase, only a small proportion of patients received iron in the catch up phase (34%). Studies by Ashworth and Nzioki et al reported lower supplementation levels of vitamins and micronutrients at Kenyatta National Hospital and South Africa [6, 7]. Feeding of malnourished children should be started early using a low osmolality and low lactose recipe, like F-75, that provides the required calories and proteins to maintain basic physiological processes. Our study showed that 92% of patients received the correct amount of F-75 in the right frequency as per the WHO recommendation. Ashworth et al showed that children with severe malnutrition were given adult meals [2].

The rehabilitation phase is heralded by return of appetite and reduction in edema. In this phase, the recommended milk based formula used was F-100 that has 100 kilocalories and 2.9g of protein per 100ml. So, frequent small feeds were prescribed initially with gradual increases to prevent complications such as cardiac failure. This was done during the transition period. Overall, the majority of children were correctly transited from F-75 to F-100 with 85% of children receiving correct volumes as required by WHO guidelines.

### Limitation of the study

Since this was the 1st study of its kind to assess the level of adherence to WHO guidelines at Garissa PGH, we were limited in terms of making comparisons to check for improvements or otherwise and therefore we could only make comparisons to studies done elsewhere. Further to this, the study was limited to assessment of the 1st 8 steps and steps 9 and 10 (supervised structured play and effective community follow-up after discharge) were not assessed in this study.

## Conclusion

This study was evaluating the level of adherence to WHO guidelines at a provincial hospital. Overall, the level of adherence was documented in five out of the eight steps and that appropriate management of children with severe acute malnutrition was inadequate at Garissa Provincial Hospital. We recommend that improving hospital infrastructure in putting up “malnutrition rooms”, improving staffing levels and training of health care workers on the implementation of WHO guideline can improve quality of care for these children.
